# Supplementary material for: Unmasking social distant damage of developed regions’ lifestyle: A decoupling analysis of the indecent labour footprint
Source: PLoS One. 2020 Apr 1;15(4):e0228649. doi: 10.1371/journal.pone.0228649 (PMC7112200; doi:10.1371/journal.pone.0228649)
Supplement: S1 Appendix — (DOCX) [file pone.0228649.s001.docx]

**Unmasking social distant damage of developed regions’ lifestyle: A decoupling analysis**

García-Alaminos, Ángela; Monsalve, Fabio; Zafrilla, Jorge; Cadarso, Maria-Angeles

**S1 Appendix. Regional and sectorial structure**

Table A in S1 Appendix. WIOD regions and codes.

| *Code* | *WIOD Region* |
| --- | --- |
| *AUS* | Australia |
| *AUT* | Austria |
| *BEL* | Belgium |
| *BGR* | Bulgaria |
| *BRA* | Brazil |
| *CAN* | Canada |
| *CHE* | Switzerland |
| *CHN* | China |
| *CYP* | Cyprus |
| *CZE* | Czech Republic |
| *DEU* | Germany |
| *DNK* | Denmark |
| *ESP* | Spain |
| *EST* | Estonia |
| *FIN* | Finland |
| *FRA* | France |
| *GBR* | United Kingdom |
| *GRC* | Greece |
| *HRV* | Croatia |
| *HUN* | Hungary |
| *IDN* | Indonesia |
| *IND* | India |
|  |  |
| *Code* | ***WIOD Region*** |
| *IRL* | Ireland |
| *ITA* | Italy |
| *JPN* | Japan |
| *KOR* | Korea |
| *LTU* | Lithuania |
| *LUX* | Luxembourg |
| *LVA* | Latvia |
| *MEX* | Mexico |
| *MLT* | Malta |
| *NLD* | Netherlands |
| *NOR* | Norway |
| *POL* | Poland |
| *PRT* | Portugal |
| *ROU* | Romania |
| *RUS* | Russia |
| *SVK* | Slovak Republic |
| *SVN* | Slovenia |
| *SWE* | Sweden |
| *TUR* | Turkey |
| *TWN* | Taiwan |
| *USA* | United States |
| *RoW* | Rest of the World |

Source: Timmer, Dietzenbacher [1]

Table B in S1 Appendix. Correspondence between the 14-sectors structure employed in the model, ISIC-Rev 3 and WIOD sectors.

| ***ISIC-Rev. 3*** | ***Sectors in the MRIO model*** | ***WIOD Sectors*** |
| --- | --- | --- |
| A. Agriculture, hunting and forestry | **Agriculture and fishing** | Crop and animal production, hunting and related service activities |
| B. Fishing |  | Fishing and aquaculture |
|  |  | Forestry and logging |
| C. Mining and quarrying | **Mining** | Mining and quarrying |
| D. Manufacturing | **Manufacturing** | Manufacture of food products, beverages and tobacco products |
|  |  | Manufacture of textiles, wearing apparel and leather products |
|  |  | Manufacture of wood and of products of wood and cork, except furniture; etc. |
|  |  | Manufacture of paper and paper products |
|  |  | Printing and reproduction of recorded media |
|  |  | Manufacture of coke and refined petroleum products |
|  |  | Manufacture of chemicals and chemical products |
|  |  | Manufacture of basic pharmaceutical products and pharmaceutical preparations |
|  |  | Manufacture of rubber and plastic products |
|  |  | Manufacture of other non-metallic mineral products |
|  |  | Manufacture of basic metals |
|  |  | Manufacture of fabricated metal products, except machinery and equipment |
|  |  | Manufacture of computer, electronic and optical products |
|  |  | Manufacture of electrical equipment |
|  |  | Manufacture of machinery and equipment n.e.c. |
|  |  | Manufacture of motor vehicles, trailers and semi-trailers |
|  |  | Manufacture of other transport equipment |
|  |  | Manufacture of furniture; other manufacturing |
|  |  | Repair and installation of machinery and equipment |
| E. Electricity, gas and water supply | **Electricity, gas and water supply** | Electricity, gas, steam and air conditioning supply |
|  |  | Water collection, treatment and supply |
|  |  | Sewerage; waste collection, treatment and disposal activities; materials recovery; etc. |
| F. Construction | **Construction** | Construction |
| G. Wholesale and retail trade; repair of motor vehicles, motorcycles and personal and household goods | **Wholesale and retail trade; repair of motor vehicles, motorcycles and personal and household goods** | Wholesale and retail trade and repair of motor vehicles and motorcycles |
|  |  | Wholesale trade, except of motor vehicles and motorcycles |
|  |  | Retail trade, except of motor vehicles and motorcycles |
| H. Hotels and restaurants | **Hotels and restaurants** | Accommodation and food service activities |
| I. Transport, storage and communications | **Transport, storage and communications** | Land transport and transport via pipelines |
|  |  | Water transport |
|  |  | Air transport |
|  |  | Warehousing and support activities for transportation |
|  |  | Postal and courier activities |
|  |  | Publishing activities |
|  |  | Motion picture, video and television programme production, sound recording and music publishing activities; etc. |
|  |  | Telecommunications |
|  |  | Computer programming, consultancy and related activities; information service activities |
| J. Financial intermediation | **Financial intermediation** | Financial service activities, except insurance and pension funding |
|  |  | Insurance, reinsurance and pension funding, except compulsory social security |
|  |  | Activities auxiliary to financial services and insurance activities |
| K. Real estate, renting and business activities | **Real estate, renting and business activities** | Real estate activities |
|  |  | Legal and accounting activities; activities of head offices; management consultancy activities |
|  |  | Architectural and engineering activities; technical testing and analysis |
|  |  | Scientific research and development |
|  |  | Advertising and market research |
|  |  | Other professional, scientific and technical activities; veterinary activities |
|  |  | Rental and leasing activities, Employment activities, Travel services, security and services to buildings |
| L. Public administration and defence; compulsory social security | **Public administration and defence; compulsory social security** | Public administration and defence; compulsory social security |
| M. Education | **Education** | Education |
| N. Health and social work | **Health and social work** | Human health and social work activities |
| O. Other community, social and personal service activities |  |  |
| P. Activities of private households as employers and undifferentiated production activities of private households | **Other services and activities** | Creative, Arts, Sports, Recreation and entertainment activities and all other personal service activities |
|  |  | Activities of households as employers; undifferentiated goods- and services-producing activities of households for own use |
| Q. Extraterritorial organizations and bodies |  | Activities of extra-territorial organizations and bodies |
| X. Not elsewhere classified |  |  |

Source: Own elaboration

**References**

1. Timmer MP, Dietzenbacher E, Los B, Stehrer R, de Vries GJ. An Illustrated User Guide to the World Input–Output Database: the Case of Global Automotive Production. Review of International Economics. 2015;23(3):n/a-n/a. doi: 10.1111/roie.12178.
