# Supplementary material for: Unmasking social distant damage of developed regions’ lifestyle: A decoupling analysis of the indecent labour footprint
Source: PLoS One. 2020 Apr 1;15(4):e0228649. doi: 10.1371/journal.pone.0228649 (PMC7112200; doi:10.1371/journal.pone.0228649)
Supplement: S2 Appendix — (DOCX) [file pone.0228649.s002.docx]

**Unmasking social distant damage of developed regions’ lifestyle: A decoupling analysis**

García-Alaminos, Ángela; Monsalve, Fabio; Zafrilla, Jorge; Cadarso, Maria-Angeles

**S2 Appendix. Social impacts database**

One of the goals of this paper is to provide information about occupational injuries and deaths and forced labour, which are some of the most troubling phenomena concerning worldwide labour markets. The quantification of social magnitudes concerning indecent labour is one of the current challenges of international labour statistics. One of the main obstacles hindering social sustainability research is the lack of data in official reports and databases –both at a sectoral and regional level- since many countries demonstrate certain reluctancy to provide sensitive information to international organisations. ILO claims that the coverage of occupational injuries might suffer from under notification and/or informal workers not having access to compensation schemes [1], so the figures that it provides for developing regions where the statistical services are poorer or informal economy has a greater dimension could be underestimated with respect to developed regions or even missing. Emerging countries are precisely the areas to highlight when social sustainability is being analysed, so the usefulness of these sources is slowed down. Additionally, the information provided by different organisms has not been compiled by any organism in order to configure a complete dataset.

In consequence, it was necessary to create a social indicators database by joining and harmonising all the available information and estimating the missing values for each of the three social indicators, aiming to achieve the level of detail required by an MRIO model following the path settled by [2]. As a result of this process, each of these indicators have been detailed for 41 regions and 18 sectors (aligned with ISIC-Rev 3) with annual frequency in the period 1996-2013. Lately, these 18 sectors have been aggregated into 14 as shown in Table B in S1 Appendix, and the time span used in this analysis has been reduced to 2000-2013. This social indicators database is published in a public repository that offers free access to the data [3].

An occupational injury is defined by the International Labour Organization [1] as “any personal injury, disease or death resulting from an occupational accident”. An occupational injury can be fatal (where death happened within one year of the day of the accident) or non-fatal (considering only those with lost work time). According to the same source, an occupational injury is different from an occupational disease, which is “a disease contracted as a result of exposure over a period of time to risk factors arising from work activity” and is not recorded in this database. Once defined non-fatal and fatal occupational injuries, the criteria followed along the generation of these indicators is summarized in Figure A in S2 Appendix. Both of these variables where generated in parallel taking as the main reference the International Labour Organization Statistics [4]. In the case of missing data for European countries, additional sources like EUROSTAT [5], the *European health for all* database [6] or even national statistics were checked. The specific series selected in EUROSTAT was chosen to ensure that road accidents on the way to work were not considered as non-European countries don’t include them in their reported data to ILOSTAT.

Concerning non-EU countries, some cases –for instance, Canada, Korea or Brazil- were not included in the baseline source. However, their national statistics services [7-10] provided detailed information that was liable to adapt to the sectoral disaggregation of interest and to the selected time span through an extrapolation process. Main obstacles have risen when seeking information for China, India and Indonesia. India and Indonesia report to the ILO unrealistic data (surprisingly, the number of fatalities and non-fatal injuries reported by India is smaller than those reported by Czech Republic), and China doesn’t provide any data to international organisms and doesn’t even mention these phenomena in their national statistics. Therefore, these three countries numbers have been estimated taking as a starting point the proposal by Hämäläinen, Takala [11] for 1998 – which was checked to be coherent with other sources like Darisman [12] in the case of Indonesia -and under the following assumptions: Indonesia’s evolution is considered to be similar to the data reported to ILOSTAT, India variables follow the same growth index as those corresponding to Sri Lanka (available in ILOSTAT [4]) and Chinese indicators growth at the same pace as the number of beneficiaries of occupational accident insurance (data available in National Bureau of Statistics of China [13]). Once total numbers have been estimated, sectoral disaggregation for these three countries is carried on following the same pattern as Mexico. Data gaps for other countries were assessed using official sources [14-19].

**Figure A in S2 Appendix. Process diagram for the generation of fatal and non-fatal injuries indicators.**

Source: Own elaboration

Regarding the forced labour indicator, the quantification of modern slavery was specially defying as it is one of the current challenges of international labour statistics, not only because it is one of the most abject phenomena in worldwide labour markets that is not usually included in official statistics, but also because there is a lack of consensus about its definition despite the efforts made by ILO in order to harmonize both the concept and the related methodology [20]. In this paper, we work under the definition of forced labour established by the ILO Forced Labour Convention No. 29 from 1930 [21] as “all work or service which is exacted from any person under the threat of a penalty and for which the person has not offered himself or herself voluntarily”, excluding sexual exploitation from our analysis since it is not attributable to any specific economic sector in the MRIO framework. The process followed along the generation of the indicator is described in Figure B in S2 Appendix.

Despite the aforementioned obstacles, some strokes have been traced by the International Labour Organization and other international Organisms [22-26], Human Rights Associations [27, 28] and academic research [29-32] to quantify this phenomenon. After a deep review of the most relevant literature concerning this issue, the starting point were two documents that allow for estimating the total of victims of forced labour in Europe in 2010, 2011 y 2012. The first of them is an article by Datta and Bales [31] that produces reliable estimations validated by the EU about the total of people suffering modern slavery (sexual and not sexual) in the EU in 2012. The second one is an official report [26] that allows for extracting the participation of sexual slavery over total victims in order to exclude it and to extrapolate Datta and Bales data to 2010 and 2011. Relying on these two sources we were able to estimate non-sexual slavery (also referred to as economic slavery in the following lines) in the EU in 2000-2013 following the process described in Figure B in S2 Appendix.

When estimating for non-EU countries, the total of slavery victims was determined according to the incidence retrieved from ILO [22] for different regions and disaggregating into countries using population. Sexual slavery was taken away from the figures either using percentages given by Lerche [29], either taking the participations in some EU country with similar socio-economic characteristics (the percentage applied to developed non-EU economies was the average value for France, Great Britan and Germany).

Once total figures of forced labour for each country and year was estimated, sectoral disaggregation was implemented according to the participation of each sector in low-skilled labour in each region provided by WIOD employment accounts. This practice is justified by two reasons. First, forced labour is characterized by several international organisms as a phenomenon happening mostly in low-skill activities such as mining, agricultural o manufacturing sectors [22, 25, 33, 34], being agriculture most probably the single largest sector in which forced labour happens [35]. This lack of skills and formation is precisely one of the determinants that most commonly generate vulnerability among workers and that makes them prone to be trapped into networks operating with modern slavery [36]. Low-skilled migrant workers, children and indigenous peoples are have among the most vulnerable collectives [37], and all of them could be considered to be low-skill workforce (children and indigenous people being forced workers commonly haven’t had proper chances to acquire medium or high skills). This pattern applies not only to developing regions: research results confirm that forced labour in the UK is located in sectors characterized by low-skilled and low-paid labour [38]. Therefore, since we found enough references linking low-skilled activities and forced labour, we reckon that providing a sectoral disaggregation based on the shares of low-skilled workers among industries is a sensible practice. Second, this disaggregation method has been already implemented by other authors when sectoral breakdown is required for indecent labour variables. Simas, Golsteijn [39] generate forced labour and child labour indicators in a similar way as we do: they split original ILO data into 163 economic sectors by using each sector’s low-skilled labour share with respect to total low-skilled labour in the region as it is can be checked in the supporting materials of their aforementioned work. Xiao, Lenzen [40] Also rely on employment accounts to generate a sectoral breakdown for data that is only provided at an aggregate level for each region.

**Figure B A in S2 Appendix. Process diagram for the generation of forced labour indicator.**


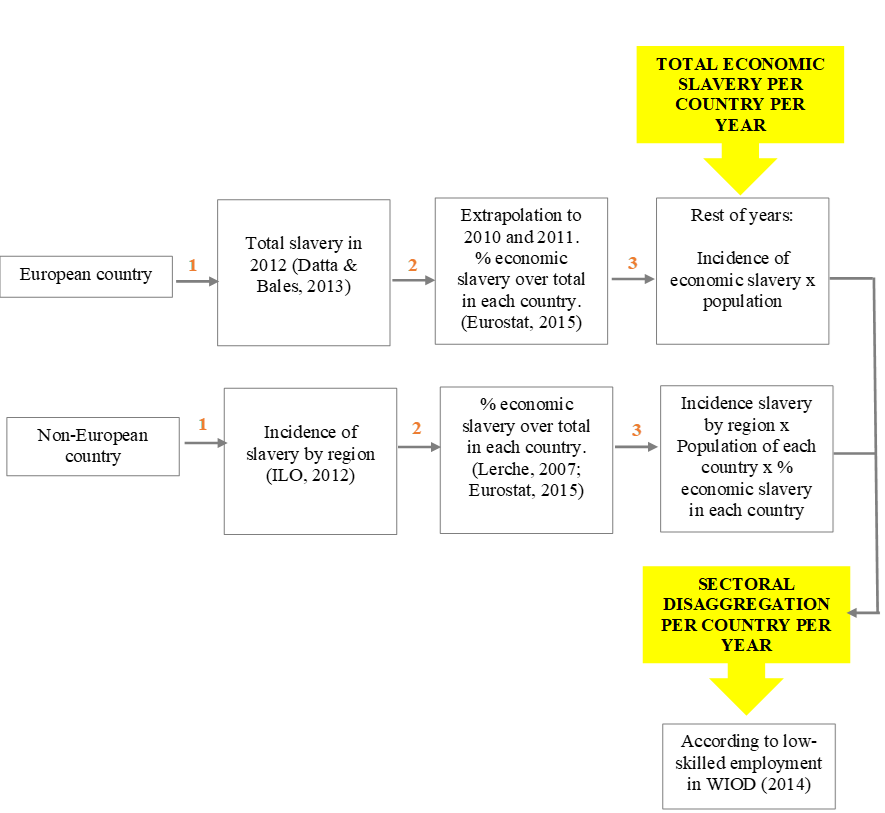


Source: Own elaboration

*Rest of the World* region (RoW) constitutes a special item. Its figures for the three indicators were estimated taking the Mexican patterns as a proxy of the average behavior of countries included in RoW. First, the incidence in Mexico of each indicator was calculated as the ratio between cases - provided by ILO [4] for fatal and non-fatal injuries and estimated as indicated in previous lines for forced labour - and hours of work - provided by WIOD [41]. Once retrieved each indicator’s incidence on each year in Mexico, they were was applied to the hours of work in RoW provided by WIOD [41] in order to obtain an estimation of total cases on each year in RoW. Sectoral disaggregation in RoW replicated the Mexican one by applying each industry’s share in Mexico over total figures estimated for RoW.

**References**

1. ILO. Decent Work and the Sustainable Development Goals: A Guidebook on SDG Labour Market Indicators. Geneva: ILO; 2018.

2. Gómez-Paredes J, Yamasue E, Okumura H, Ishihara KN. The labour footprint: A framework to assess labour in a complex economy. Economic Systems Research. 2015;27(4):1-25. doi: 10.1080/09535314.2014.998173.

3. García-Alaminos A. Social Indicators of Working Conditions Database. 1 ed. Mendeley Data2019.

4. Main statistics. Occupational injuries [Internet]. International Labour Organization. 1996-2013 [cited 18/07/2016]. Available from: <http://www.ilo.org/ilostat/>.

5. Health and safety at work statistics [Internet]. European Commission. 1996-2013 [cited 31/08/2016]. Available from: <http://ec.europa.eu/eurostat/data/database>.

6. European health for all database [Internet]. World Health Organization. Regional Office for Europe. 1996-2013 [cited 20/07/2016]. Available from: <http://data.euro.who.int/hfadb/>.

7. Statistics Canada's key socioeconomic database (CANSIM) [Internet]. Sattistics Canada. 1996 [cited 09/12/2016].

8. Detailed Key Statistical Measures Report [Internet]. AWCC. 1996-2011 [cited 07/12/2016].

9. Base de dados históricos de acidentes do trabalho [Internet]. Previdência Social e INSS. 1999-2013 [cited 14/07/2016]. Available from: <http://www3.dataprev.gov.br/aeat/>.

10. Industrial Accident Statistics. [Internet]. Korean Statistical Information Service, Government of the Republic of Korea. 1996-2013 [cited 07/15/2016].

11. Hämäläinen P, Takala J, Saarela KL. Global estimates of occupational accidents. Safety Science. 2006;44(2):137-56.

12. Darisman M. OSH Status Report for Indonesia. Bandung: Local Iniciative for OSH Network, 2011.

13. China Statistical Yearbook. Basic Statistics of Social Insurance. [Internet]. NBS. 1995-2013 [cited 25/07/2016]. Available from: <http://www.stats.gov.cn/tjsj/ndsj/2013/indexeh.htm>.

14. Industrial Accidents Statistics in Japan. T [Internet]. Ministry of Health, Labor and Welfare. 2009-2010 [cited 07/15/2016].

15. Statistics on accidents and illnesses at work [Internet]. Secretariat of Labor and Social Welfare 2014 [cited 07/28/2016].

16. Social and Economic Indicators of the Russian Federation [Internet]. Federal State Statistics Service. 1991-2014 [cited 07/27/2016].

17. Social Indicators [Internet]. Directorate-General of Budget, Accounting and Statistics. 2007, 2013 [cited 07/27/2016].

18. Occupational Injuries and Illnesses and Fatal Injuries Profiles [Internet]. U.S. Department of Labor. 2011 [cited 07/19/2016].

19. Social Conditions, Accidents at Work [Internet]. Statistics Portal Luxembourg. 1996-2010 [cited 07/20/2016].

20. ILO. Guidelines concerning the measurement of forced labour. Geneva: 20th International Conference of Labour Statisticians, 2018.

21. ILO. Forced Labour Convention (No. 29). Geneva: 1930.

22. ILO. Global Estimate of Forced Labour. Geneva: International Labour Organization, 2012.

23. ILO. A global alliance against forced labour : global report under the follow-up to the ILO Declaration on Fundamental Principles and Rights at Work 2005 / International Labour Conference, 93rd session 2005. Geneva: International Labour Organization, 2005 9221153606.

24. ILO. Stopping forced labour : global report under the follow-up to the ILO Declaration on fundamental principles and rights at work. Geneva, Switzerland: 2001.

25. USBILA. List of Goods Produced by Child Labor or Forced Labor. Washington, D.C.: United States Bureau of International Labor Affairs, 2016.

26. EUROSTAT. Trafficking in human beings. Luxemburgo: European Union, 2015.

27. Walk Free Foundation. Tackling Modern Slavery in Supply Chains: A Guide 1.0. Broadway Nedlands, Australia: 2015.

28. Walk Free Foundation. Global Slavery Index. Broadway Nedlands, Australia: Walk Free Foundation, 2014.

29. Lerche J. A Global Alliance against Forced Labour? Unfree Labour, Neo-Liberal Globalization and the International Labour Organization. Journal of Agrarian Change. 2007;7(4):425-52. doi: 10.1111/j.1471-0366.2007.00152.x.

30. Hernandez D, Rudolph A. Modern day slavery: What drives human trafficking in Europe? European Journal of Political Economy. 2015;38:118-39. doi: <http://doi.org/10.1016/j.ejpoleco.2015.02.002>.

31. Datta MN, Bales K. Slavery in Europe: part 1, estimating the dark figure. Human Rights Quarterly. 2013;35(4):817-29.

32. Fletcher LE, Bales K, Stover E. Hidden slaves: Forced labor in the United States. Berkeley Journal of International Law. 2005;23(1):47-96.

33. Fletcher LE, Bales K, Stover E. Hidden slaves: Forced labor in the United States. 2005.

34. Srivastava RS. Bonded Labor in India: Its Incidence and Pattern. Geneva: ILO, 2005.

35. Belser P. Forced Labour and Human Trafficking: Estimating the Profits. SSRN Electronic Journal. 2005. doi: 10.2139/ssrn.1838403.

36. ILO. Strengthening action to end forced labour. International Labour Conference 103rd Session 2014; Geneva, Switzerland2014.

37. ILO. Stopping forced labour and slavery-like practices - The ILO strategy. Geneva, Switzerland2012.

38. Geddes A, Craig G, Scott S, Ackers L, Robinson O, Scullion D. Forced labour in the UK. Bristol: JRF Programme Paper, 2013.

39. Simas M, Golsteijn L, Huijbregts M, Wood R, Hertwich E. The “Bad Labor” Footprint: Quantifying the Social Impacts of Globalization. Sustainability. 2014;6(11):7514. PubMed PMID: doi:10.3390/su6117514.

40. Xiao Y, Lenzen M, Benoît-Norris C, Norris GA, Murray J, Malik A. The Corruption Footprints of Nations. Journal of Industrial Ecology. 2017:n/a-n/a. doi: 10.1111/jiec.12537.

41. Timmer MP, Dietzenbacher E, Los B, Stehrer R, de Vries GJ. An Illustrated User Guide to the World Input–Output Database: the Case of Global Automotive Production. Review of International Economics. 2015;23(3):575-605. doi: <https://doi.org/10.1111/roie.12178>.
