# Supplementary material for: Unmasking social distant damage of developed regions’ lifestyle: A decoupling analysis of the indecent labour footprint
Source: PLoS One. 2020 Apr 1;15(4):e0228649. doi: 10.1371/journal.pone.0228649 (PMC7112200; doi:10.1371/journal.pone.0228649)
Supplement: S3 Appendix — (DOCX) [file pone.0228649.s003.docx]

**Unmasking social distant damage of developed regions’ lifestyle: A decoupling analysis**

García-Alaminos, Ángela; Monsalve, Fabio; Zafrilla, Jorge; Cadarso, Maria-Angeles

**S3 Appendix. Worldwide decoupling (footprint perspective): detailed results**

Table A in S3 Appendix. Detailed results by country for worldwide decoupling (footprint perspective).

|  |  | **Positive** | | | **Negative** | | | **Coupling** |
| --- | --- | --- | --- | --- | --- | --- | --- | --- |
|  |  | Strong | Weak | Recessive | Strong | Weak | Expansive |  |
| **Fatal injuries** | 2000-2008 | AUT, BGR, CHE, CHN, DEU, ESP, EST, FRA, HUN, IND, ITA, JPN, KOR, LTU, MEX, MLT, PRT, RUS, TWN, USA | AUS, BEL, BRA, CAN, CYP, CZE, DNK, FIN, GBR, GRC, HRV, IDN, IRL, LUX, LVA, NLD, NOR, POL, ROU, SVK, SVN, SWE, ROW | - | - | TUR | - | - |
|  | 2008-2013 | AUS, BRA, CAN, CHE, CHN, IND, JPN, LUX, MEX, MLT, NOR, SVK, SWE, TWN, USA, ROW | AUT, BEL, BGR, CYP, CZE, DEU, DNK, ESP, EST, FIN, FRA, GBR, GRC, HRV, HUN, IRL, ITA, KOR, LTU, LVA, MEX, NLD, POL, PRT, ROU, RUS, SVN | AUT, BEL, BGR, CYP, CZE, DEU, DNK, ESP, EST, FIN, FRA, GBR, GRC, HRV, HUN, IRL, ITA, KOR, LTU, LVA, MEX, NLD, POL, PRT, ROU, RUS, SVN | TUR | - | - | IDN |
| **Non-Fatal Injuries** | 2000-2008 | DEU, IDN, IND, ITA, JPN, MLT, PRT, SVN, TWN, USA | AUS, AUT, BEL, BGR, CAN, CHE, CHN, CYP, CZE, DNK, ESP, EST, FRA, GBR, GRC, HRV, HUN, KOR, LTU, LUX, NLD, NOR, POL, SVK, SWE | - | - | - | BRA, LVA, MEX, ROU | FIN, IRL, RUS, ROW |
|  | 2008-2013 | CAN, IND, JPN, LUX, NOR, SVK, SWE, TWN, USA | AUS, CHE, CHN, IDN, LTU | AUT, BEL, BGR, BRA, CYP, CZE, DEU, DNK, ESP, FIN, FRA, GBR, GRC, HRV, HUN, ITA, POL, PRT, ROU, SVN | MEX, NLD, RUS, TUR | - | - | EST, IRL, KOR, MLT, ROW |
| **Forced Labour** | 2000-2008 | BGR, CHN, JPN, MLT, TWN | AUS, AUT, BEL, BRA, CHE, CYP, CZE, DEU, DNK, ESP, EST, FRA, GBR, GRC, HRV, HUN, IDN, IND, IRL, ITA, KOR, LTU, LVA, NLD, NOR, POL, PRT, ROU, RUS, SVK, SWE, USA | - | TUR | - | LUX, MEX, SVN | CAN, FIN, ROW |
|  | 2008-2013 | JPN, SVK, TWN, USA | BRA, CAN, CHN, IDN, SWE | BEL, BGR, DEU, DNK, ESP, GRC, ITA, KOR, LVA, POL, PRT | AUT, LTU, MEX, NLD, RUS, TUR | IRL, ROU, SVN | IND, LUX, MLT, ROW | AUS, CHE, CYP, CZE, EST, HRV, HUN, NOR |

Source: Own elaboration according to Vehmas, Malaska [1] and Tapio [2] classification.

*Note to Table A in S3 Appendix. This table provides further detail to the results plotted in Figure 2 in the main text.*

**References**

1. Vehmas J, Malaska P, Luukkanen J, Kaivo-oja J, Hietanen O, Vinnari M, et al. Europe in the global battle of sustainability: Rebound strikes back?–Advanced Sustainability Analysis. Publications of the Turku School of Economics and Business Administration, Series Discussion and Working Papers. 2003;7:2003.

2. Tapio P. Towards a theory of decoupling: degrees of decoupling in the EU and the case of road traffic in Finland between 1970 and 2001. Transport Policy. 2005;12(2):137-51. doi: <http://dx.doi.org/10.1016/j.tranpol.2005.01.001>.
