# Supplementary material for: Unmasking social distant damage of developed regions’ lifestyle: A decoupling analysis of the indecent labour footprint
Source: PLoS One. 2020 Apr 1;15(4):e0228649. doi: 10.1371/journal.pone.0228649 (PMC7112200; doi:10.1371/journal.pone.0228649)
Supplement: S4 Appendix — (DOCX) [file pone.0228649.s004.docx]

**Unmasking social distant damage of developed regions’ lifestyle: A decoupling analysis**

García-Alaminos, Ángela; Monsalve, Fabio; Zafrilla, Jorge; Cadarso, Maria-Angeles

**S4 Appendix. Worldwide decoupling analysis based on PBA measure**

The analysis of worldwide decoupling following PBA criteria is shown in Figures A, B and C in S4 Appendix. Please notice that elasticities are now obtained by using value added (VA) as the economic driving force of social impacts. When the same analysis was done in footprint terms, the economic indicator chosen as the driving force was Gross National Expenditure (GNE) since a consumption point of view was being applied.

Our results show a clear difference between occupational injuries and forced labour. Concerning fatal and non-fatal injuries, there is a notorious dispersion among countries. However, most regions achieved the goal of social decoupling in both periods analyzed (see Figures B and C in S4 Appendix). In these two indicators, the predominant typologies of decoupling are strong and weak positive in the expansive period 2000-2008 and recessive decoupling from 2008 onwards. Comparing both indicators, positive decoupling is more solid in the case of fatal injuries as there are more regions in the strong area than in the weak and decoupling zones.

Looking at the time evolution of decoupling concerning forced labour generated domestically, Figure A in S4 Appendix shows that it worsens its performance along time: while in the expansive period countries most countries were placed in the weak and positive decoupling areas, in the recessive period there has been a massive displacement towards strong and weak negative decoupling. It is interesting to notice that forced labour is the only indicator that depicts worse results for domestic measures than for the footprint ones. One reason behind this difference between the domestic and the footprint perspective might be the increasing involvement of developing countries in global value chains, which may be reducing strong positive decoupling in the first period and fostering strong negative decoupling in the second in PBA terms with respect to CBA terms.

**Figure A** **in S4 Appendix. Domestic forced labour worldwide decoupling. Worldwide data (44 regions). 2000-2008 and 2008-2013.**

**
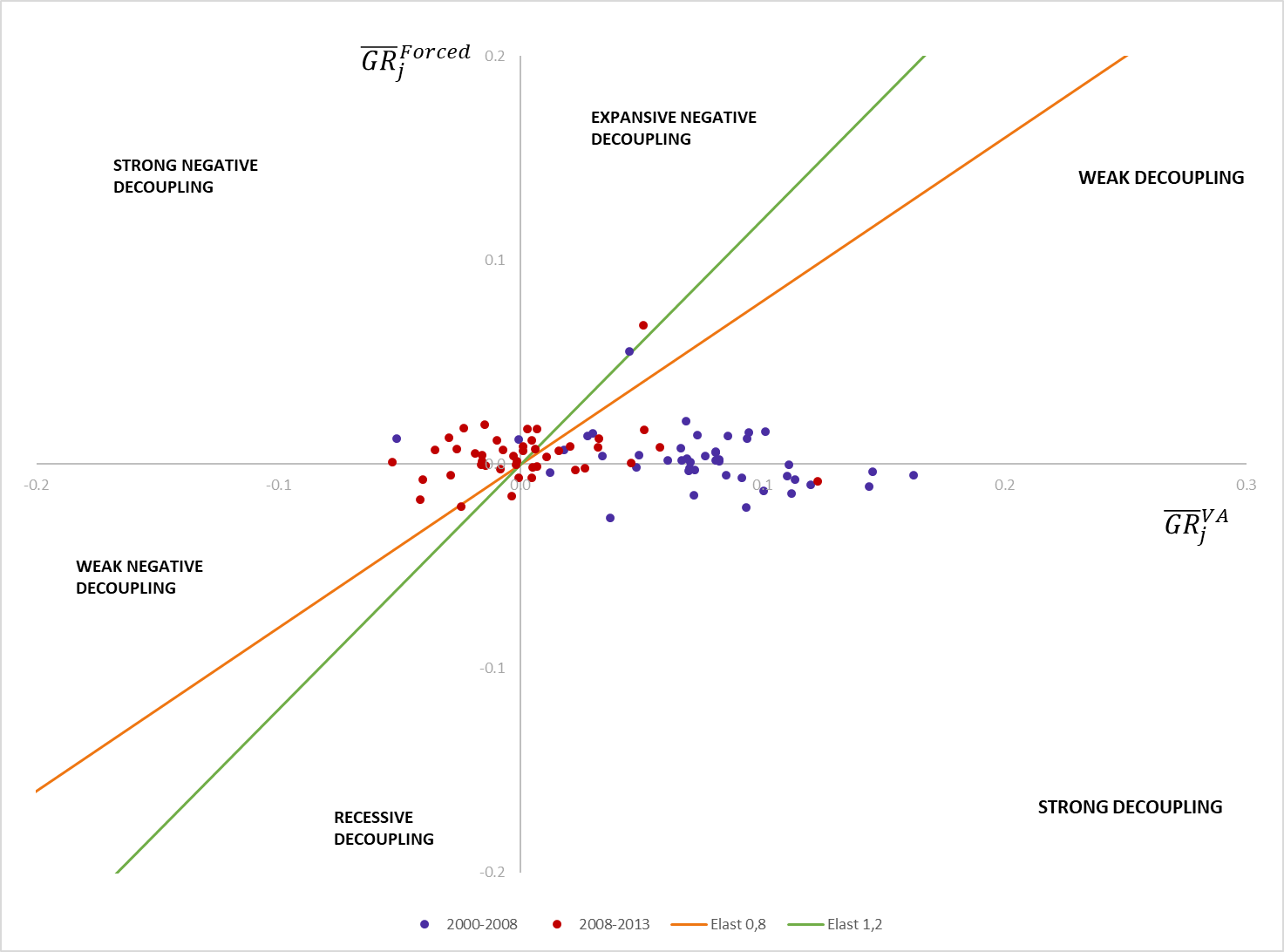
**Source: Own elaboration based on Timmer, Dietzenbacher [1] and Timmer, Los [2].

*Note to Figure A in S4 Appendix. The vertical axis represents the average growth rate of forced labour on each of the two periods plotted, while the horizontal axis represents the average growth rate of VA on each of these two periods. The social indicators used in the calculus of elasticities are expressed in PBA terms. For further information about the typologies of decoupling, please check Fig 1 in the main text.*

**Figure B in S4 Appendix. Domestic fatal injuries worldwide decoupling. Worldwide data (44 regions). 2000-2008 and 2008-2013.**


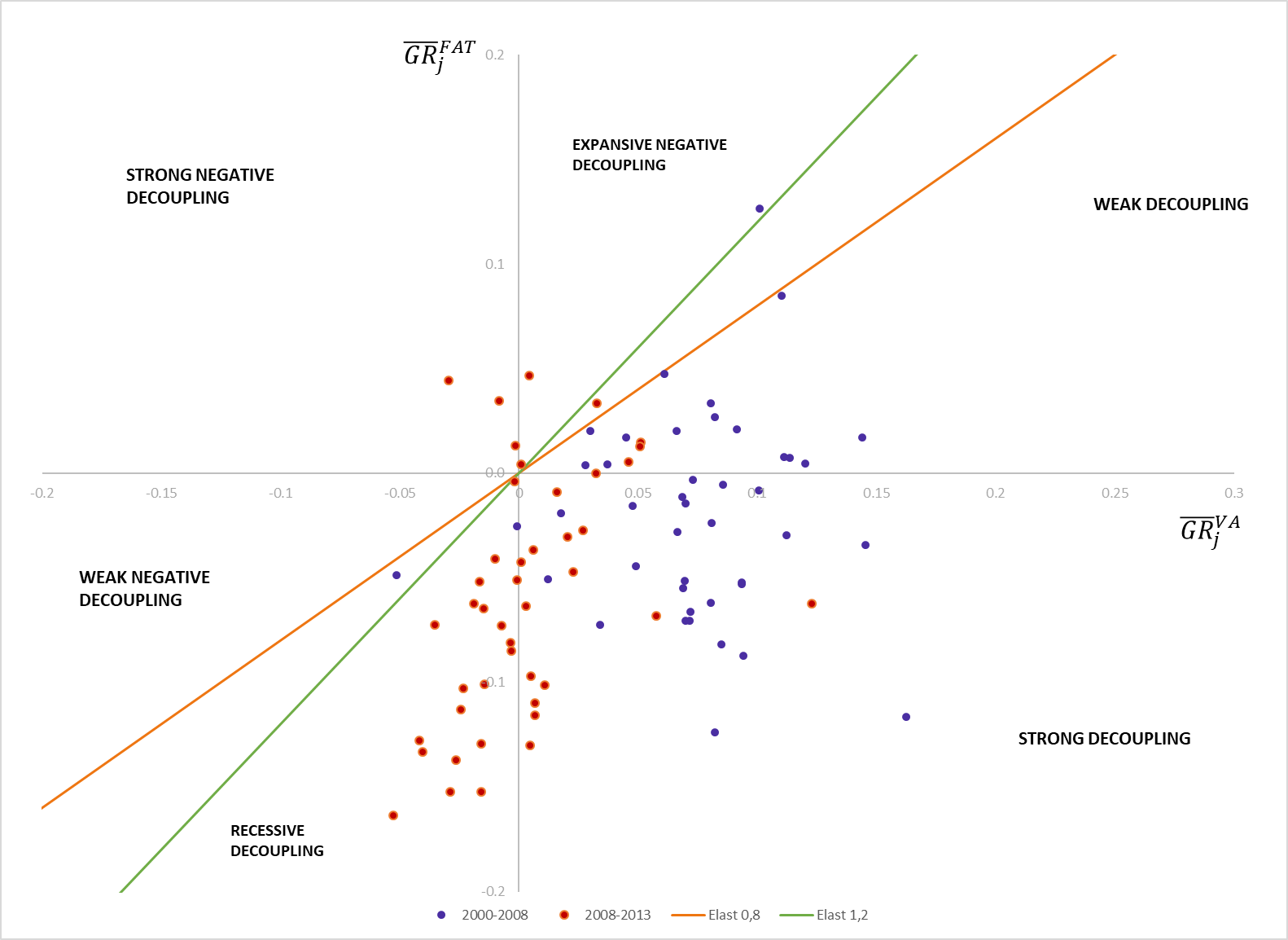


Source: Own elaboration based on Timmer, Dietzenbacher [1] and Timmer, Los [2].

*Note to Figure B in S4 Appendix. The vertical axis represents the average growth rate of fatal injuries on each of the two periods plotted, while the horizontal axis represents the average growth rate of VA on each of these two periods. The social indicators used in the calculus of elasticities are expressed in PBA terms. For further information about the typologies of decoupling, please check Fig 1 in the main text.*

**Figure C in S4 Appendix. Domestic non-fatal injuries worldwide decoupling. Worldwide data (44 regions). 2000-2008 and 2008-2013.**


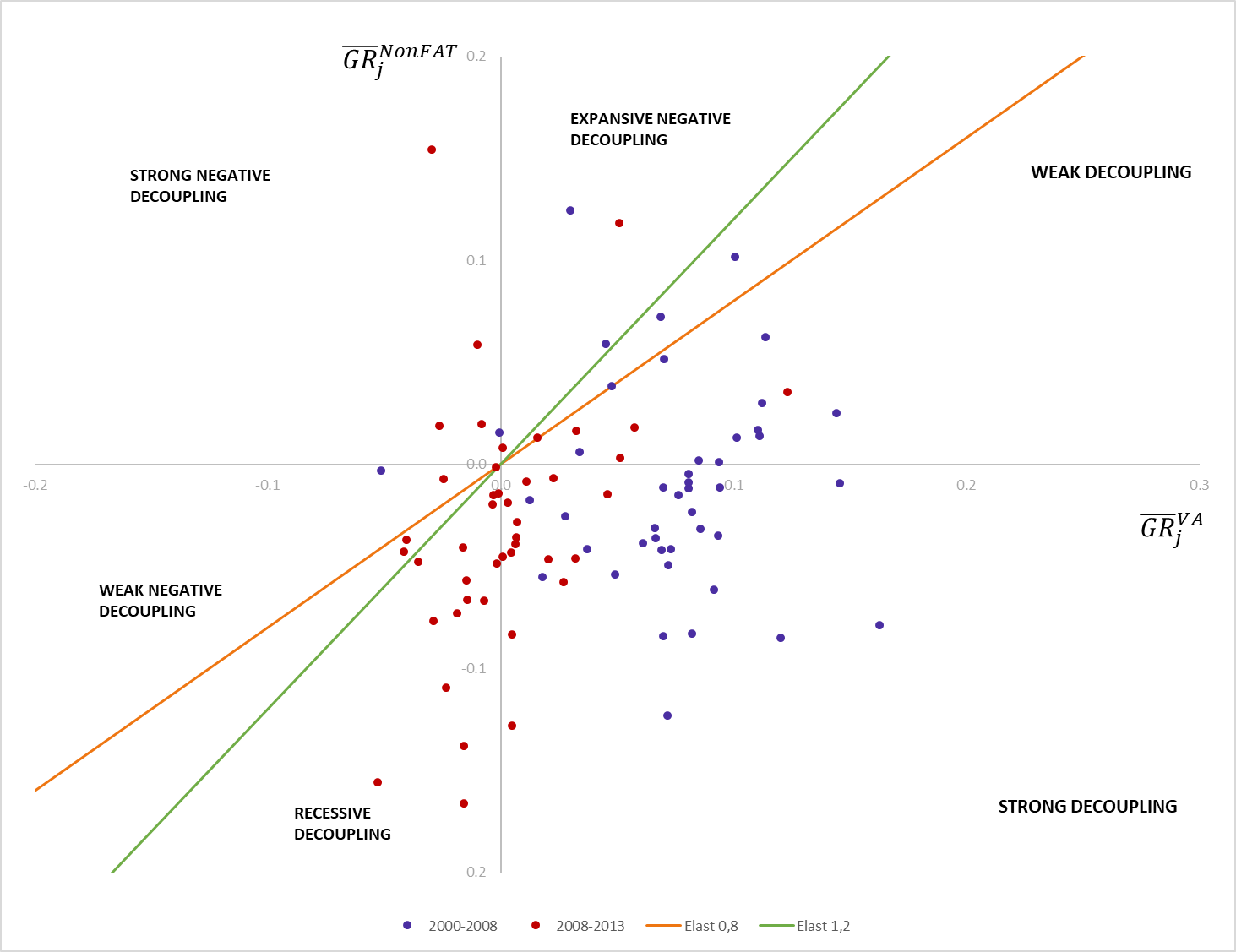


Source: Own elaboration based on Timmer, Dietzenbacher [1] and Timmer, Los [2].

*Note to Figure C in S4 Appendix. The vertical axis represents the average growth rate of non-fatal injuries on each of the two periods plotted, while the horizontal axis represents the average growth rate of VA on each of these two periods. The social indicators used in the calculus of elasticities are expressed in PBA terms. For further information about the typologies of decoupling, please check Fig 1 in the main text.*

**References**

1. Timmer MP, Dietzenbacher E, Los B, Stehrer R, de Vries GJ. An Illustrated User Guide to the World Input–Output Database: the Case of Global Automotive Production. Review of International Economics. 2015;23(3):575-605. doi: <https://doi.org/10.1111/roie.12178>.

2. Timmer MP, Los B, Stehrer R, de Vries GJ. An anatomy of the global trade slowdown based on the WIOD 2016 release. Groningen Growth and Development Centre (GGDC) Research Memorandum. 2016;(162).
