# Supplementary material for: Unmasking social distant damage of developed regions’ lifestyle: A decoupling analysis of the indecent labour footprint
Source: PLoS One. 2020 Apr 1;15(4):e0228649. doi: 10.1371/journal.pone.0228649 (PMC7112200; doi:10.1371/journal.pone.0228649)
Supplement: S5 Appendix — (DOCX) [file pone.0228649.s005.docx]

**Unmasking social distant damage of developed regions’ lifestyle: A decoupling analysis**

García-Alaminos, Ángela; Monsalve, Fabio; Zafrilla, Jorge; Cadarso, Maria-Angeles

**S5 Appendix. Preliminary decoupling analysis**

In this preliminary analysis, we present the evolution of both the footprint of the social indicators of interest and the Gross National Expenditure (GNE) made by the region (Figures A and B in S5 Appendix). In these figures social decoupling is indicated by labour indicator lines running below the GNE line (grey line). The comparison between the evolution of the footprint indicators and the domestic ones

**Figure A in S5 Appendix. Fatal injuries, non-fatal injuries and forced labour footprints and real GNE. EU-28 (upper part, A) and USA (lower part, B). Relative changes 2000-2013.**

**
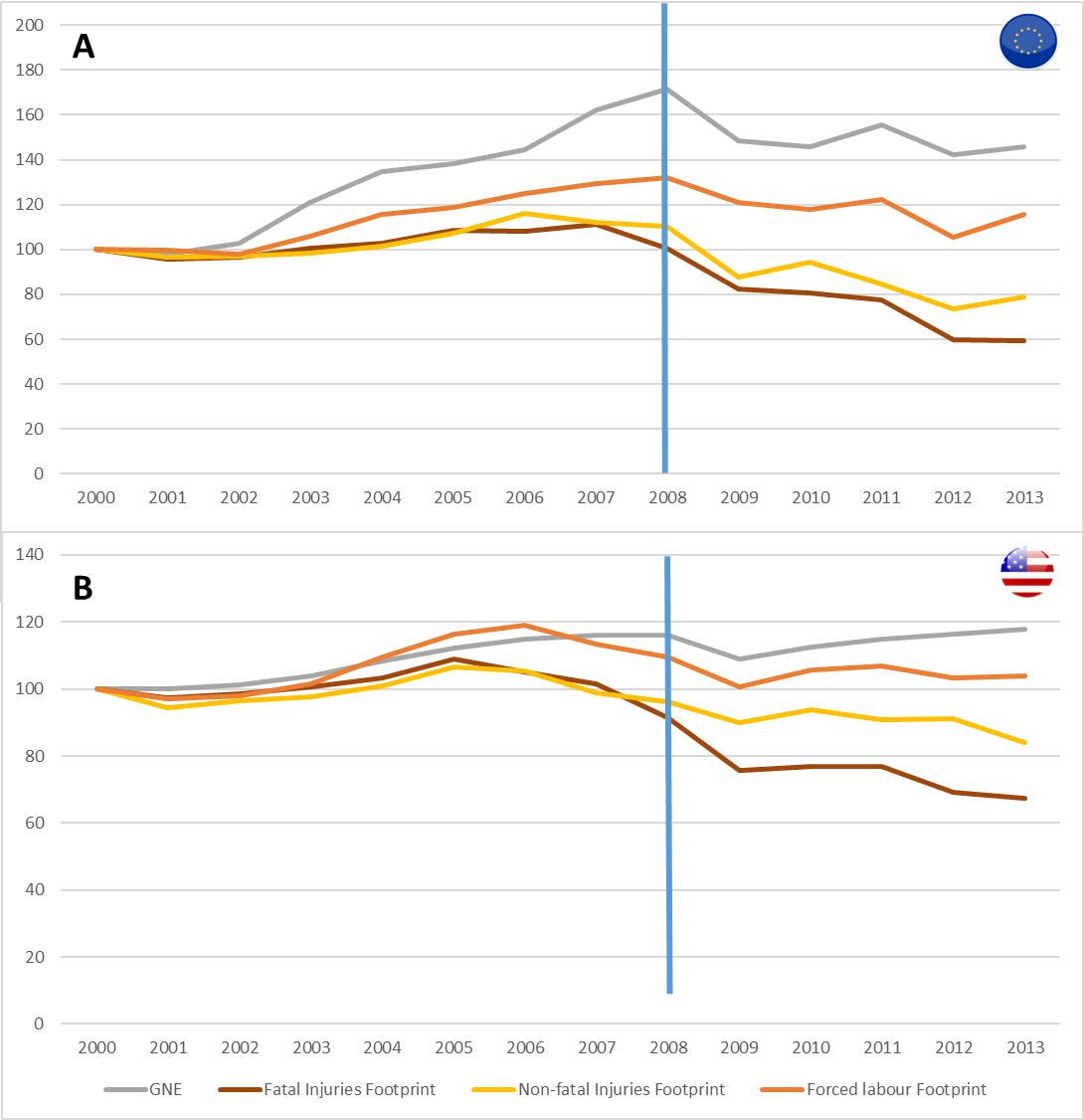
**

Source: Own elaboration

*Note to Figure A in S5 Appendix. Panel A stands for EU figures while panel B provides USA figures. Each one of the variables is represented in terms of relative changes taking 2000 as base year.*

Starting by the EU-28, the upper part in Figure A in S5 Appendix shows that GNE sets two different periods to analyze: 2000-2008, where an economic expansive path was followed by this region, and 2008-2013, where GNE in real terms declined and oscillated around similar levels than those achieved in 2007. In the expansive period, fatal and non-fatal injuries and forced labour footprint grew too but at a lower rate than GNE, so we can talk about relative but not absolute decoupling in terms of these three impacts. From 2008 onwards, when GNE fell and oscillated, the evolution of the social impacts footprint was diverse: fatal injuries footprint declined faster, which imply talking about recessive decoupling in terms of Vehmas, Malaska [1] classification. In this second period, non-fatal injuries and forced labour footprints slightly declined, but it is unclear if this fall in the footprint was caused just by the recessive shock or by an improvement in labour conditions in the regions supplying Europe.

Looking at the lower part in Figure A in S5 Appendix, GNE in the United States (USA) suffered an eventual decline in 2008-2009, but after that, it started again its growth path. Fatal injuries footprint suffered relative decoupling until 2005, year in which this magnitude started an intense fall while GNE was still growing. Therefore, absolute decoupling could be claimed to be happening in this variable since 2005. On the other hand, non-fatal injuries and forced labour footprints exhibited a soft increase until 2005, which could be a sign of relative decoupling. After 2005, they unsteadily declined remaining almost stable, which corresponds to an absolute decoupling process.

Figure B in S5 Appendix shows the evolution of both the footprint of the social indicators of interest and the GNE made by the region. In these figures, social decoupling is indicated by labour indicator lines running below the GNE line (grey line).

**Figure B in S5 Appendix. Fatal injuries, non-fatal injuries and forced labour domestic cases and real GNE. EU-28 (upper part, A) and USA (lower part, B). Relative changes 2000-2013.**


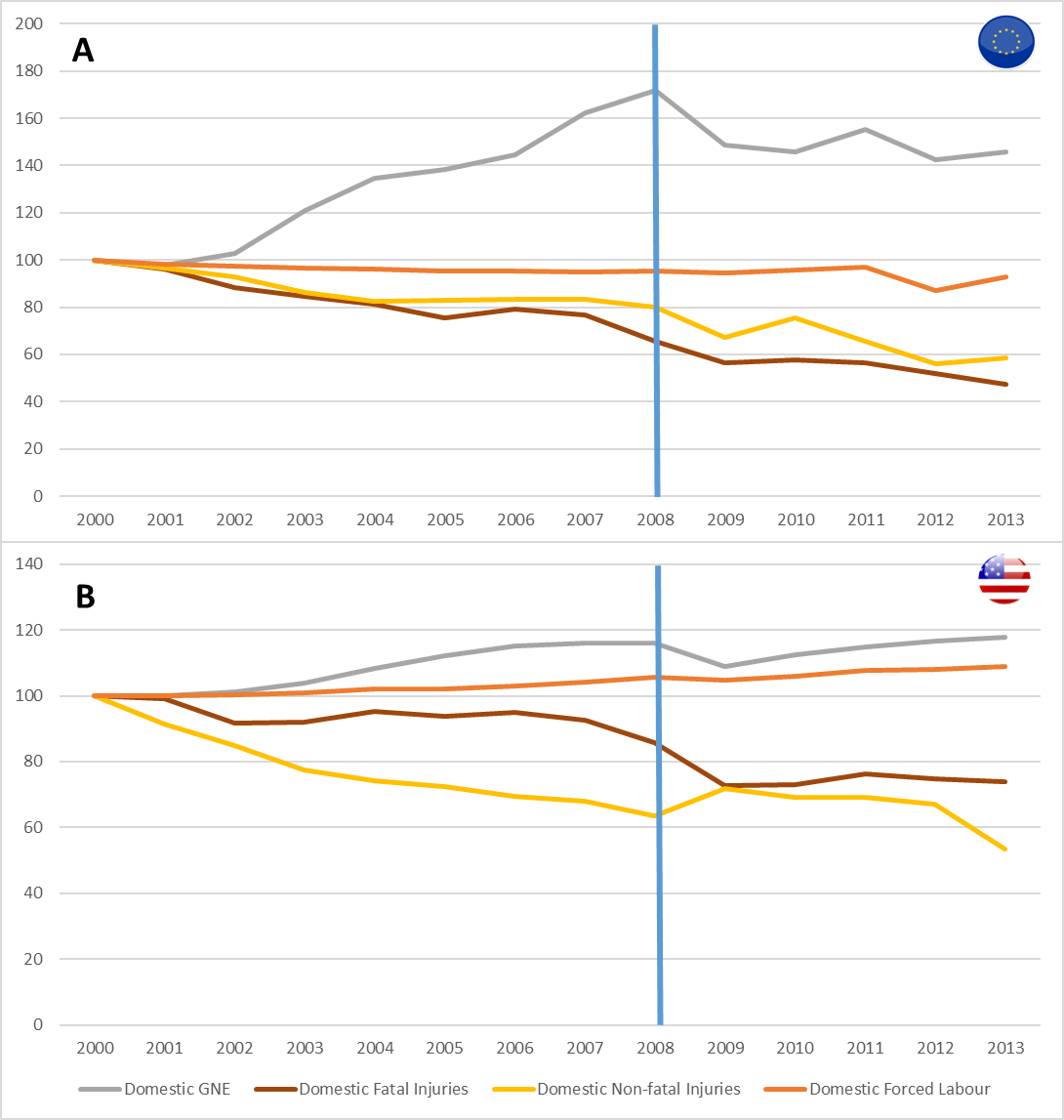


*Note to Figure B in S5 Appendix. Panel A stands for EU figures while panel B provides USA figures. Each one of the variables is represented in terms of relative changes taking 2000 as base year.*

According to the upper part in Figure B in S5 Appendix, the EU-28 clearly showed absolute decoupling in the first period for the three impacts assessed since fatal and non-fatal injuries and forced labour figures generated inside EU-28 fell or remain stable while GNE grew. In the recessive period from 2008 onwards, GNE fell and oscillated while fatal and non-fatal injuries kept falling faster than GNE, which shows recessive decoupling. In the case of forced labour, it seemed to remain stable during this second period until the last years in which it fell. Therefore, forced labour in terms of PBA seems to be decoupling too in the second period but at a lower extent than occupational injuries.

Focusing on the USA at the lower part in Figure B in S5 Appendix, absolute decoupling is outstanding in both kinds of occupational injuries along the two periods considered: both indicators fell notoriously while GNE kept growing. On the other hand, forced labour in USA grew at a lower rate than GNE along the whole time expand, so relative decoupling is identified for this indicator.

**References**

1. Vehmas J, Malaska P, Luukkanen J, Kaivo-oja J, Hietanen O, Vinnari M, et al. Europe in the global battle of sustainability: Rebound strikes back?–Advanced Sustainability Analysis. Publications of the Turku School of Economics and Business Administration, Series Discussion and Working Papers. 2003;7:2003.
